# GJA1/CX43 High Expression Levels in the Cervical Spinal Cord of ALS Patients Correlate to Microglia-Mediated Neuroinflammatory Profile

**DOI:** 10.3390/biomedicines10092246

**Published:** 2022-09-10

**Authors:** Nunzio Vicario, Paola Castrogiovanni, Rosa Imbesi, Sebastiano Giallongo, Giuliana Mannino, Debora Lo Furno, Rosario Giuffrida, Agata Zappalà, Giovanni Li Volti, Daniele Tibullo, Michelino Di Rosa, Rosalba Parenti

**Affiliations:** 1 Department of Biomedical and Biotechnological Sciences, Section of Physiology, School of Medicine, University of Catania, 95125 Catania, Italy; 2 Department of Biomedical and Biotechnological Sciences, Section of Anatomy, Histology and Movement Sciences, School of Medicine, University of Catania, 95125 Catania, Italy; 3 Department of Biomedical and Biotechnological Sciences, Section of Biochemistry, School of Medicine, University of Catania, 95125 Catania, Italy; 4 Department of Chemical, Biological, Pharmaceutical and Environmental Sciences, University of Messina, 98166 Messina, Italy

**Keywords:** GJA1, ALS, brain, bioinformatic, microglia

## Abstract

Amyotrophic lateral sclerosis (ALS) is a progressive neurodegenerative disorder affecting motoneurons (MNs) with a fatal outcome. The typical degeneration of cortico-spinal, spinal, and bulbar MNs, observed in post-mortem biopsies, is associated with the activation of neuroimmune cells. GJA1, a member of the connexins (Cxs) gene family, encodes for connexin 43 (Cx43), a core gap junctions (GJs)- and hemichannels (HCs)-forming protein, involved in cell death, proliferation, and differentiation. Recently, Cx43 expression was found to play a role in ALS pathogenesis. Here, we used microarray and RNA-seq datasets from the NCBI of the spinal cord of control (NDC) and ALS patients, which were stratified according to the GJA1 gene expression. Genes that positively or negatively correlated to GJA1 expression were used to perform a genomic deconvolution analysis (GDA) using neuroimmune signatures. Expression analysis revealed a significantly higher GJA1 expression in the MNs of ALS patients as compared to NDC. Gene deconvolution analysis revealed that positively correlated genes were associated with microglia activation, whereas negatively correlated genes were associated with neuronal activation profiles. Moreover, gene ontology analysis, performed on genes characterizing either microglia or neuronal signature, indicated immune activation or neurogenesis as main biological processes. Finally, using a synthetic analysis of drugs able to revert the GJA1 transcriptomic signatures, we found a specific drug profile for ALS patients with high GJA1 expression levels, composed of amlodipine, sertraline, and prednisolone. In conclusion, our exploratory study suggests GJA1 as a new neuro-immunological gene correlated to microglial cellular profile in the spinal cord of ALS patients. Further studies are warranted to confirm these results and to evaluate the therapeutic potential of drugs able to revert typical GJA1/CX43 signature in ALS patients

## 1. Introduction

Inflammation and reactive glial activation are common hallmarks of degenerative disease [1]. Thus, understanding the overall biological mechanisms underlying triggering factors and potential strategies to modulate glial phenotype are of significant importance in this field [1]. Amyotrophic lateral sclerosis (ALS) is a progressive neurodegenerative disease affecting upper and lower motoneurons (MNs) [1]. Whether loss of function is a direct consequence of neuronal loss, their susceptibility and vulnerability are closely linked to resident glial cell and immune cell activation and neurotoxic potential [1,2].

Neuroglial cross-talk in the central nervous system (CNS) microenvironment are on the spotlight given their contribution to either neuronal suffering and degeneration or, vice versa, as an exploitable biological tool to counteract chronicization and disease progression, favouring either self or exogenous regenerative mechanisms [3]. Intercellular communication and ion channels establish a delicate balance of fundamental importance to maintain tissue homeostasis in adult life and to coordinate patterning during development, whereby their dysfunction irreversibly leads to functional impairment and cell suffering [4]. Disruption of such a delicate balance irreversibly leads to functional impairment and cell suffering, particularly in the CNS [5]. Recent evidence supports the hypothesis of a significant involvement of connexins (CXs)-based channels in the progression and chronicization of inflammatory and degenerative diseases, including multiple sclerosis (MS), Alzheimer’s disease (AD), Parkinson’s disease (PD), and amyotrophic lateral sclerosis (ALS) [6,7,8,9,10,11]. Exchanges of small molecules, metabolites and energy substrates are guaranteed and regulated by gap junctions (GJs) and hemichannels (HCs), composed by CXs, which form transmembrane pores between adjacent cells or between the intracellular and extracellular compartment, respectively [5]. CXs are the core GJs and HCs forming proteins. Six units of CXs form a HC and two docked HCs form a GJ. Such structures are usually aggregated in defined plasma membrane areas called plaques, rapidly assembled and remodelled [5]. In the CNS, GJs and HCs would exert significant effects on neuronal function, astrocytes reactive activation, microglia inflammatory polarization, and neurovascular modulation [12,13,14,15].

A plethora of data have defined the role of CXs signature of glial cell populations to critically influence the working microenvironment of neurons, affecting neuronal homeostasis and plasticity [16]. Astrocytes mainly express CX43, encoded by GJA1, largely involved in maintaining intercellular cross-talk and energy metabolism in physiological conditions, whereas it has been found to sustain degenerative and reactive chronic astroglial activation in pathological conditions [16,17]. Although to a lower level, astrocytes also express CX30, encoded by GJB6, and CX26, encoded by GJB2, critically linked to GJs and HCs formation in degeneration and neurotoxic conditions [18,19]. Microglia, the resident myeloid inflammatory cells of the CNS, express CXs depending on their resting or activated profile [20]. In particular, CX43 plays an important role in mediating heterocellular coupling with astrocytes or neurons [21]. It has been reported that CX43 increase associated with high levels of CX32, encoded by GJB1, and CX36, encoded by GJD2, play a role in the inflammatory polarization of microglia and in the activation of neurotoxic signals [22,23,24]. In fact, in similar conditions, the increase of CX32- and CX43-HCs, allowing the release of neurotoxic levels of glutamate and ATP, modifies the composition of the CNS microenvironment, inducing inflammatory and/or degenerative conditions [23,25,26]; at the same time, CX36, mainly forming heterocellular GJs with neurons, may represent a mechanism for transferring death signals from microglia to neurons [27]. In particular, we recently reported a leading role of either CX43-based HCs and CX43-based GJs in modulating the intercellular network in CNS, thus influencing the operating nervous environment [28,29].

Herein, we used a microarray datasets-based analysis of human brain biopsies from the spinal cord of ALS patients to correlate GJA1 expression and ALS progression and pathological features. Our analysis also aimed at uncovering GJA1 biological functions in ALS pathogenesis and the involved biological signalling pathways correlating with higher GJA1 expression levels in the ALS spinal cord.

## 2. Materials and Methods

### 2.1. Daset Selection

The NCBI Gene Expression Omnibus (GEO) database (http://www.ncbi.nlm.nih.gov/geo/, 5 September 2022) was used to select transcriptome datasets of interest [30]. Mesh terms “amyotrophic lateral sclerosis”, “spinal cord”, and “human” were used to identify the potential datasets to select. We sorted the datasets by the number of samples (high to low), and by the clinical data. The datasets were selected following the criteria exposed in the section “Clinical and pathological criteria”. We selected two datasets showed in Table 1.

### 2.2. Sample Stratification

Microarray datasets from biopsies of NDC and of ALS patients were collected.

As reported by the original authors of the GSE26927, the dataset was composed of 19 dissected snap-frozen cervical spinal cord (<100 mg) of control not ALS affected (NDC = 9) and affected by sporadic Amyotrophic lateral sclerosis (ALS = 10). Complete experimental details are available in the reference publication [31].

Regarding the GSE76220, we obtained the data from RNA-sequencing (RNA-seq) belonging to ALS gene-expression signatures in laser capture micro-dissected (LCM) MNs from post-mortem spinal cords. LCM of spinal cord sections from 10 sporadic ALS patients and 9 controls (NDC) was performed, total RNA was extracted and RNA-seq libraries were sequenced on Illumina GA II platform [32]. Furthermore, by stratifying the ALS patients from GSE26927 according to GJA1 expression levels, as a subset signature, we found that 2542 unique genes (including GJA1) were significantly positively correlated (GSPC) (r-range from 0.50 to 0.94) and 3110 unique genes were significantly negatively correlated (GSNC) (r-range from −0.50 to −0.96) to GJA1 expression levels (Table 2 and supplementary Table S1).

### 2.3. Clinical and Pathological Criteria

Analyzed samples coming from spinal cord biopsies were collected immediately after surgery and stored at −80 °C until use. Sample selection was made by original authors taking into account sample pH and RNA integrity number (RIN). All patients included in this study signed an informed consent and the study was approved by the medical ethics committees of all sites [31,32].

### 2.4. Data Processing and Experimental Design

A MultiExperiment Viewer (MeV) software (The Institute for Genomic Research (TIGR), J. Craig Venter Institute, La Jolla, CA, USA) was used to process and identify Significantly Different Expressed Genes (SDEG) within the datasets. When multiple gene probes were found with the same GeneID NCBI, those with the highest variance were used.

With the aim of identifying genes commonly modulated between the GSE datasets present in Table 1 and cell type-specific genes for brain cells, we performed a Venn diagram analysis, using the web-based utility Venn Diagram Generator (http://bioinformatics.psb.ugent.be/webtools/Venn/, 5 September 2022) [33,34]. For the microarray dataset, we also performed a statistical analysis with GEO2R, applying a Benjamini–Hochberg false discovery rate test [35,36,37].

Gene ontology (GO) analysis was performed using the web utility GeneMANIA (http://genemania.org/, 5 September 2022) [38], STRING (https://string-db.org/, 5 September 2022) [39], and the GATHER (Gene Annotation Tool to Help Explain Relationships) (http://changlab.uth.tmc.edu/gather/, 5 September 2022) [40]. The GeneMANIA was also used for building the weighted gene networks commonly modulated, rendered by CorelDRAW2020 (Corel Corporation, Ottawa, ON, Canada). CIRCOS was used to plot the GO analysis (http://mkweb.bcgsc.ca/tableviewer/, 5 September 2022) [41].

Additionally, publicly available RNA-seq data were used to carry out a panel of cell-tissue-specific genes; namely, astrocytes (n = 191), endothelial cells (endotheliocytes) (n = 76), neurons (n = 1032), microglia (n = 118), and oligodendrocytes (n = 111) [42]. From GSE46236, we sorted the SDEG of pericyte inflammatory (n = 333) [43].

Furthermore, we decided to deepen the analysis including the immune system cellular profiles, consisting of classical natural killer (NK) (n = 125), CTLs (n = 62), T helper cell type 1 (Th1) (n = 221) and 2 (Th2) (n = 98), obtained from the GSE22886 and two populations of macrophages, classical and alternative activated, macrophages M1 (n = 823) and macrophages M2 (n = 160) from GSE5099. The immune-cell GSE22886 dataset was composed by isolated 12 different types of human leukocytes from peripheral blood and bone marrow. In order to obtain the genes characterizing these cells, we have excluded all significant genes in common between all types of human leukocytes and successively, we selected only genes that were mutually and exclusively significantly up-regulated [34,44].

The web utility AmiGO 2 (http://amigo.geneontology.org/amigo/landing, 5 September 2022) [45,46] was used to define genes closely linked to the process synaptic transmission genes (n = 45) (GO:0099564). In order to identify genes involved in this process, results were filtered using the following parameters: organism, mammalian-type of sources, gene-evidence type, experimental.

Lastly, in an effort to broaden the results of our analysis, we downloaded data related to different biological processes from the Kyoto Encyclopedia of Genes and Genomes (KEGG) database: host–virus interaction (279), inflammatory response (704), and Synaptic Vesicle Cycle (78). KEGG is a database that contains information related to the biological system, such as cell, organism, and ecosystem, from molecular-level information. We selected the following biological processes: cholinergic synapse, and synaptic vesicle cycle (https://www.genome.jp/kegg/, 5 September 2022) [47,48,49].

### 2.5. Drugs Analysis Prediction (DAP)

To identify potential pharmacological agents for ALS patients according to sex and GJA1 expression levels, we used the L1000fwd, Large Scale Visualization of Drug Induced Transcriptomic Signatures web-based utility (https://maayanlab.cloud/L1000FWD/, 5 September 2022) [50]. L1000fwd calculates the similarity between an input gene expression signature and the LINCS-L1000 data, in order to rank drugs potentially able to reverse the transcriptional signature [50]. An adjusted *p*-value (q-value) of 0.05 has been considered as threshold for statistical significance. Combined score (CS) is calculated by multiplying the logarithm of the *p*-value from the Fisher exact test and the Z-score as a composite index:

C=z×log10 (p)

### 2.6. Statistical Analysis

Statistical analysis was performed using Prism 9 software (GraphPad Software, La Jolla, CA, USA). Exact hypergeometric probability was used to calculate statistically significant differences of intersection between 2 lists of genes. The representation factor (RF) shows whether genes from one list (list A) are enriched in another list (list B), assuming that genes behave independently. The RF is defined as (number of genes in common between both lists) (number of genes in the genome)/(number of genes in list A) (number of genes in list B). RF > 1 indicates more overlap than expected between the two independent groups, a RF < 1 indicates less overlap than expected, and a RF of 1 indicates that the two groups are identical by the number of genes expected to be independent in the groups.

The representation factor=x÷expected # of genes

Expected # of genes=(n×D)÷N

The probability of finding x overlapping genes can be calculated using the hypergeometric probability formula:

Probability of overlapping=C(D, x)×C(N−D, n−x)÷C(N,n)

where x = # of genes in common between two groups; n = # of genes in group 1; D = # of genes in group 2; N = total genes, in this case 20,203 genes (RefSeq, a database run by the US National Center for Biotechnology Information (NCBI)); C (a,b) is the number of combinations of a thing taken ‘b’ at ‘a’ time [51,52].

Ordinary one-way Analysis of Variance (ANOVA) followed by Tukey’s multiple comparisons test was used to assess statistical differences between groups and correlations were assessed using Pearson correlation test. Differences between groups were considered as statistically significant with adjusted *p* value < 0.05.

All data used in the present study were transformed for the analysis in Z-score intensity signal. Z score was constructed by taking the ratio of weighted mean difference and combined standard deviation according to Box and Tiao (1992) [53].

The efficiency of each biomarker was assessed by the receiver operating characteristic (ROC) curve analyses. Nonparametric ROC curves analyzed NDC versus ALS. The area under the ROC curve (AUC) and its 95% confidence interval indicates diagnostic efficiency. The accuracy of the test with the percent error is reported [54].

## 3. Results

### 3.1. High Expression Levels of GJA1 in MNs of ALS Patients Compared to NDC

**Figure 1:**
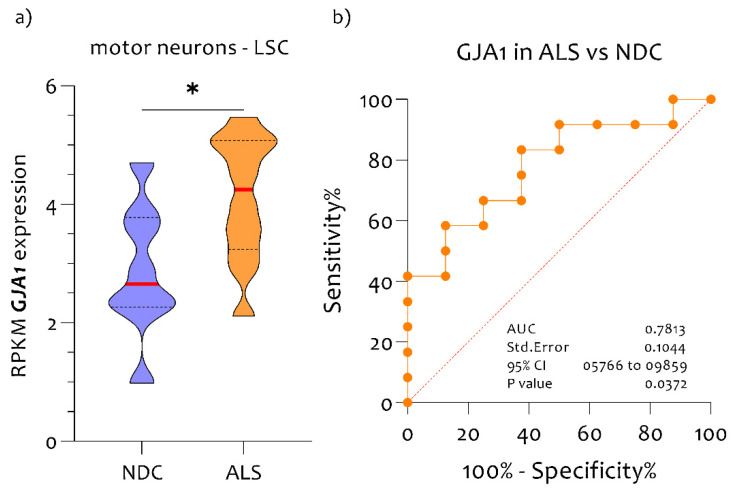
GJA1 is highly expressed in the spinal cord and MNs of ALS patients compared to NDC. (**a**) GJA1 expression levels in MNs of NDC (n = 9) and ALS (n = 13); (**b**) perfect diagnostic ability of GJA1 expression levels to discriminate NDC subjects from ALS patients (AUC = 0.7813, *p* < 0.037). Data are expressed as RPKM intensity expression levels (means and SD) and presented as violin dot plots. * *p*-value < 0.01.

GJA1 expression analysis on LCM dissected MNs from post-mortem spinal cords revealed a significant increase in RNA expression levels in ALS patients compared to NDC subjects (*p* = 0.023) (Figure 1a). To evaluate the potential diagnostic ability of GJA1 to discriminate between ALS patients to NDC subjects, we applied a receiver operating characteristic (ROC) analysis. GJA1 expressed a good diagnostic ability to discriminate NDC from ALS in spinal cord (AUC = 0.7813, *p* = 0.037) (Figure 1b).

### 3.2. ALS Patients Exhibit Different Neuro-Immune Cellular Profile According to GJA1 Expression Levels

We conducted a genomic deconvolution analysis (GDA) using neuro-immune signatures obtained from GEO Datasets, KEGG, and AmiGo (Table 3). Cell signatures covered five neurological, seven immune cells populations, and five biological processes, as described in the Section 2.

**Figure 2:**
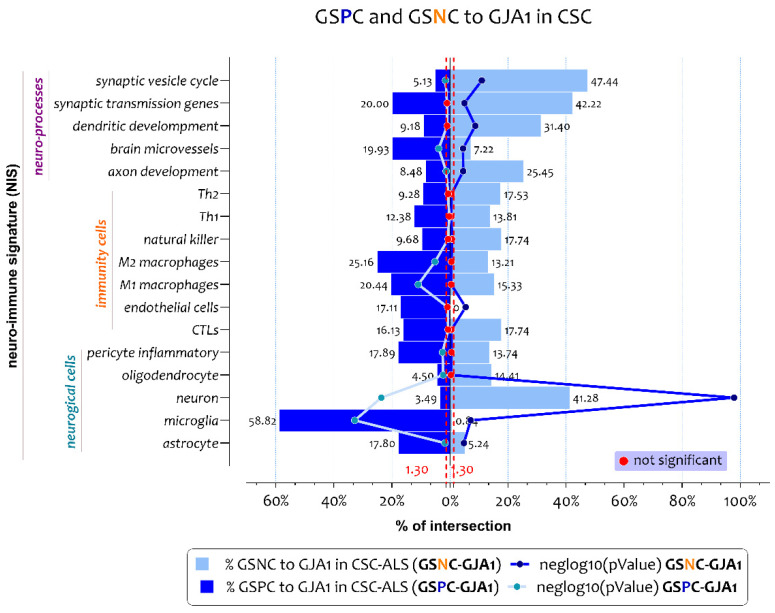
Neuro-immune signature deconvolution analysis obtained by GSPC/GSNC-GJA1 in the spinal cord of ALS patients. The intersection of gene lists positively/negatively (GSPC/GSNC) correlated to GJA1 expression levels in ALS patients to the 17 neuro-immune signatures belonging to the CNS cells (n = 5), the immune cells (n = 7), and biological processes (n = 5). The genes in common obtained from the overlaps are expressed in percentages and represented as a bar chart.

We intersected the gene lists that characterize the neuro-immune signatures to the gene lists significantly positively and negatively correlated to GJA1 expression levels in spinal cord of ALS patients (Figure 2 and supplementary Table S1).

**Figure 3:**
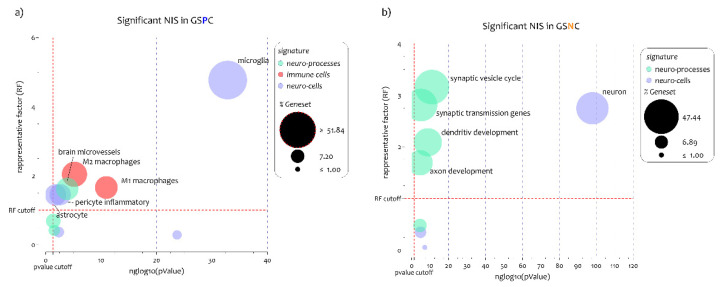
Significant intersection between neuro-immune signature and GSPC/GSNC-GJA1 in the spinal cord of ALS patients. (**a**,**b**) The intersection of gene lists positively ((**a**), GSPC) and negatively ((**b**), GSNC) correlated to GJA1 expression levels in ALS patients to the 17neuro-immune signature belonging to the CNS cells (n = 5), the immune cells (n = 7), and biological processes (n = 5). The genes in common obtained from the overlaps are expressed in percentages and represented as the size of bobble chart. The *p*-value obtained by Fisher’s test is expressed as neglog10 (*p*-value) (abscissa axis). *p*-values < 0.05 were considered as statistically significant (neglog10 (*p*-value) > 1.30). The representation factor is reported to the ordinate axis. A RF > 1 indicates more overlap than expected between the two independent groups, a RF < 1 indicates less overlap than expected, and a RF of 1 indicates that the two groups are identical by the number of genes expected to be independent in the groups.

Neuro-immune signature-weighted GDA showed that genes positively correlated to the GJA1 expression levels (GSPC-GJA1) were closely related to the neuro-immune inflammatory profile (Figure 2). In particular, the list of GSPC-GJA1 significant intersections with neuroimmune signatures were highlighted for the cellular signatures of the microglia (n gene = 70, n% = 58, neglog10 (*p*-value) = 32.83, RF = 4.77), astrocyte (n gene = 34, n% = 17.80, neglog10 (*p*-value) = 1.76, RF = 1.44), pericyte inflammatory (n gene = 56, n% = 17.89, neglog10 (*p*-value) = 2.59, RF = 1.45), M1-macrophages (n gene = 168, n% = 20.43, neglog10 (*p*-value) = 10.92, RF = 1.65), M2-macrophages (n gene = 40, n% = 25.15, neglog10 (*p*-value) = 5.11, RF = 2.40), and brain microvessels (n gene = 58, n% = 19.83, neglog10 (*p*-value) = 3.87, RF = 1.61) (Figure 2, Figure 3a and supplementary Table S1). Regarding the excluded neuro-immune signature, such as oligodendrocyte, neuron, axon development, and synaptic vesicle cycle, the RF value was < 1, so the intersections differed significantly from the individual processes (Figure 2, Figure 3a and supplementary Table S1).

We performed a neuro-immune signature-weighted GDA using the genes negatively correlated to GJA1 expression levels (GSNC-GJA1) in the spinal cord of ALS patients (Figure 2 and supplementary Table S1). The analysis highlighted that negatively correlated genes to the GJA1 expression levels delineated a neuro-protective profile. In particular, we showed significative intersections for the signatures of neuron (n gene = 486, n% = 41.27, neglog10 (*p*-value) = 97.85, RF = 2.74), axon development (n gene = 57, n% = 25.44, neglog10 (*p*-value) = 4.51, RF = 1.69), dendritic development (n gene = 65, n% = 31.40, neglog10 (*p*-value) = 8.70, RF = 2.08), synaptic transmission genes (n gene = 19, n% = 42.22, neglog10 (*p*-value) = 4.99, RF = 2.80), and synaptic vesicle cycle (n gene = 37, n% = 47.43, neglog10 (*p*-value) = 10.92, RF = 3.15) (Figure 2, Figure 3b and supplementary Table S1). Regarding the excluded neuro-immune signature, such as astrocyte, microglia, endothelial cells, and brain microvessels, the RF value was < 1, so the intersections differed significantly from the individual processes (Figure 2, Figure 3b and supplementary Table S1).

### 3.3. Biological Processes Identified by the Genes Belonging to Microglia and Neuron Signatures Determined by GJA1 Expression Levels in the Spinal Cord of ALS Patients

**Figure 4:**
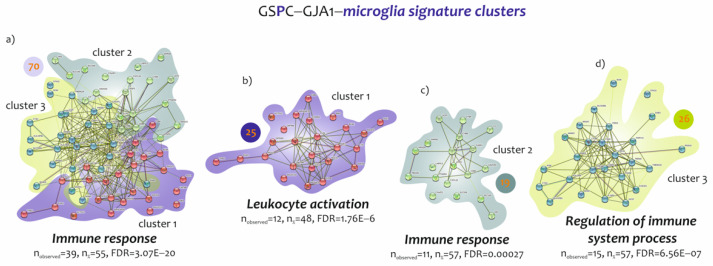
GO analysis of common genes identified by the microglia signatures determined by GJA1 high spinal cord expression levels. (**a**) Fisher t-test analysis showed 70 genes significant in common between microglia–neuro-immune signature and GSPC-GJA1 signature. The main biological process involved in activation of 70 genes was the immune response (FDR = 3.07E−20, n = 39). (**b**) Cluster 1 identified by 25 genes with the leukocyte activation as the main biological process (FDR = 1.76E−06, n = 12). (**c**) Cluster 2 identified by 19 genes with the immune response as the main biological process (FDR = 0.00027, n = 11). (**d**) Cluster 3 identified by 26 genes with the regulation of immune system process as the main biological process (FDR = 6.56E−07, n = 15).

Our results highlighted two distinct profiles that characterize ALS patients, closely associated with GJA1 expression levels in the spinal cord MNs. The profiles that emerged were related to microglia and neurons, the most regulated by GJA1 expression levels. According to these results, we decided to perform a GO analysis on genes that characterized the two highlighted signatures. Microglia cells were identified by a signature of 118 genes, of which 70 were found to be positively correlated with GJA1 expression levels. It was possible to divide the 70 genes into three different clusters, each characterized by a specific biological function (Figure 4). GO analysis on the 70 genes (59% of signature) showed that 55% (39 genes) were involved in biological processes of immune response (FDR = 3.07E−20) (Figure 4a and supplementary Table S1). Regarding the cluster 1, it consisted of 25 genes whose expression was closely related to leukocyte activation (n = 12, n = 48%, FDR = 1.76E−06) (Figure 4b). Cluster 2 was composed of 19 genes belonging to biological processes related to response to immune response (n = 11, n = 57%, FDR = 0.00027) (Figure 4c), while cluster 3, composed of 26 genes, highlighted the regulation of the immune-system process as the main biological process (n = 15, n = 57%, FDR = 6.56E−07) (Figure 4d and supplementary Table S1).

**Figure 5:**
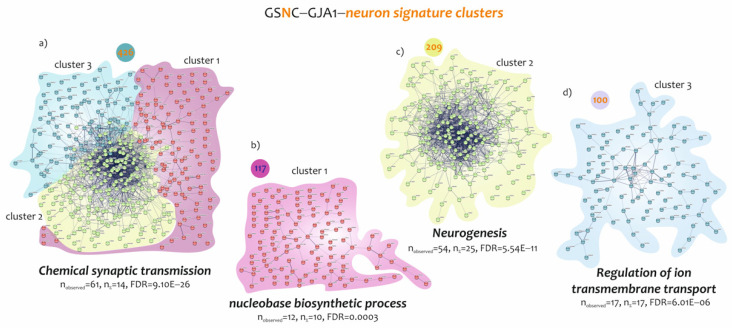
GO analysis of common genes identified by the neuron signatures determined by GJA1 low spinal cord expression levels. (**a**) Fisher t-test analysis showed 426 genes significant in common between neuron–neuro-immune signature and GSNC-GJA1 signature. The main biological process involved in activation of 426 genes was the chemical synaptic transmission (FDR = 9.10E−26, n = 61). (**b**) Cluster 1 identified by 117 genes with the nucleobase biosynthetic process as the main biological process (FDR = 0.0003, n = 12). (**c**) Cluster 2 identified by 209 genes with the neurogenesis as the main biological process (FDR = 5.54E−11, n = 54). (**d**) Cluster 3 identified by 100 genes with the regulation of ion transmembrane transport as the main biological process (FDR = 6.01E−06, n = 17).

Neurons were identified by a signature of 1032 genes, of which 426 were found to be closely negatively correlated with GJA1 expression levels. Moreover, for this signature, it was possible to divide the 426 genes into three different clusters, each characterized by a specific biological function (Figure 5). The 426 gene cluster showed as mainly modulated biological processes the chemical synaptic signalling (n = 61, n = 14%, FDR = 9.10E−26) (Figure 5a and supplementary Table S1). Regard cluster 1, consisted of 117 genes whose expression was closely related to nucleobase biosynthetic process (n = 12, n = 10%, FDR = 0.0003) (Figure 5b and supplementary Table S1). Cluster 2 consisted of 209 genes belonging to biological processes related to neurogenesis (n = 54, n = 25%, FDR = 5.54E−11) (Figure 5c), and cluster 3, composed of 100 genes, highlighted G-protein signalling regulation of ion transmembrane transport as the main biological process (n = 17, n = 17%, FDR = 6.01E−06) (Figure 5d and supplementary Table S1).

### 3.4. Effect of Drugs Mimicking and Opposing GJA1 Transcriptomic Signatures Obtained from Spinal Cord of ALS Patients

Anti-signature perturbation analysis was performed using the GSPC-GJA1 and GSNC-GJA1 signatures identified for ALS patients and uploaded to L1000FWD. Among the predicted drugs, we only highlighted those that are already in clinical use (Table 4).

**Figure 6:**
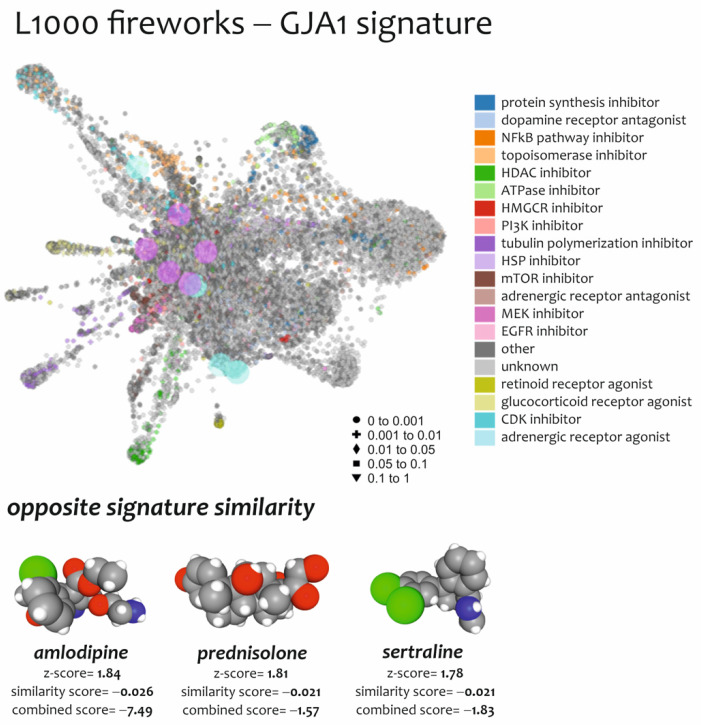
Anti-signature perturbation analysis. L1000FDW visualization of drug-induced signature. Input genes are represented by the significantly GSPC-GJA1 and GSNC-GJA1. Dots are color-coded based on the Mode of Action (MOA) of the respective drug. The drugs with a higher CS (i.e., amlodipine, sertraline, and prednisolone) were selected.

We chose to list in Table 4 the potential anti-ALS drugs identified by L1000FWD analysis using the GSPC/GSNC-GJA1 signature model. The complete list can be retrieved in supplementary Table S1. Among them: amlodipine (CS = −7.49), a second-generation calcium channel blocker that is used in the therapy of hypertension and angina pectoris; sertraline (CS = −1.83), a selective serotonin reuptake inhibitor (SSRI) used in the therapy of depression, anxiety disorders and obsessive–compulsive disorder and prednisolone (CS = −1.57), a glucocorticoid receptor agonist, used in allergies, inflammatory conditions, autoimmune disorders, and cancers (Figure 6, Table 4, and supplementary Table S1).

## 4. Discussion

Public transcriptome dataset analysis has been extensively used for the identification of novel pathogenic pathways and therapeutic targets in a number of human pathologies [55,56], including neurodegenerative diseases [33,34,57,58] and cancer [59,60]. Meta-analysis of available datasets allows the improvement of statistical power and to obtain differentially expressed gene signatures, and also estimates heterogeneity [61]. This possibility has further sparked the omic approach, which is a formidable tool for the overall assessment of pathological features, allowing pathways to be highlighted that are synergistically involved with molecular factors, and therefore new therapeutic targets towards personalized therapies [62].

The delicate balance of the CNS cell populations, together with their cross-talk, relies on CXs-based channel exchanges that maintain homeostasis controlling ions composition, trophic factors, energy substrates, and removal of waste products [63,64,65]. Neurodegenerative diseases, including MNs diseases, affect brain and/or spinal cord resident cell populations and, among these, ALS is of particular relevance due to the negative outcome and the mostly unknown etiopathology [66]. GJA1/Cx43 expression and its role as GJs- or HCs-forming protein has been investigated in preclinical models of ALS and in human biopsies [16,67,68]. In a previous study, using a selective MNs depletion model, we observed a significant increase in intercellular coupling mediated by GJA1/Cx43 between astrocytes and microglial cells in the ventral horns of the spinal cord [28]. Whether a correlation between GJA1/Cx43 and reactive astrogliosis was observed, the biological pathways involved in the potential glial cells modulation deserved further investigation.

In the present study, we analyzed human spinal cord biopsies of ALS patients to investigate the involvement of GJA1 in ALS pathogenesis and disease progression. We found significantly different GJA1 expression levels in MNs of ALS patients as compared to NDC, supporting the diagnostic potential of GJA1. Accordingly, we recently showed that spinal MNs depletion induces a robust gliosis, resulting in the reactive Cx43 expression, which hamper plasticity and spontaneous compensatory processes [28].

Gene deconvolution analysis performed on the spinal cord of ALS patients’ transcriptome, revealed that genes, positively correlated to GJA1 expression levels, were associated with activation of microglia related to neuro-immune inflammation. Accordingly, the main stimuli influencing the expression of Cx43 are the cytokines including TNF, IL-1β, IFN-c, IL-6, and NO, released following microglial activation [69,70]. Microglia, being the immune effector cells of the CNS, contributes significantly to neuroinflammation by increased levels of the HCs- and GJs-forming CX43 [64]. Indeed, inflammatory microglia via CXs-based channels allows the activation of the cell signalling pathway inducing stimulation of glutaminase and glutamate induced excitotoxicity, IL-1β release and increased extracellular ATP levels, contribute to creating degenerative signalling for neurons and induce reactive astrogliosis [71].

Our analysis also revealed that genes, negatively correlated to GJA1 expression, were associated with neuronal activation profiles, as revealed by the significative intersections with axon development, dendritic development, synaptic transmission genes, and synaptic vesicle cycle. This observation is in line with extensive vacuolation, cytoarchitectural disintegration, reduced numbers of synapses, and an apical dendrite degeneration of Betz cells, which characterize ALS [72]. Moreover, our analysis showed that neurons were identified by a signature of genes mainly clustered in biological processes related to neurogenesis. Although it is well known that CX43 protein itself regulates key signalling pathways during development and neurogenesis [73], its role in modulating synaptic plasticity and compensatory processes in neurodegenerative disease is not fully elucidated [74,75,76]. Data herein described report four main neuronal biological processes that negatively correlate with GJA1 levels, namely axon development, dendritic development, synaptic transmission genes, and synaptic vesicle cycle. This finding is of critical importance, particularly in ALS, given the evidence of potential compensatory mechanisms in neurodegenerative disease [3,77]. Indeed, immune-modulatory and antioxidant treatment for degenerative disease, including ALS, relies on endogenous compensation that involves resident stem cell pool or spared MNs [78,79,80]. In fact, once endogenous regenerative potential or compensatory mechanisms are overwhelmed, neurodegeneration and functional loss ensues [81]. Our data are in accordance with previous published literature reporting a robust MNs degeneration in the early phase of the disease, which is compensated by spared MNs. Such a compensatory process represents the biological bases for an early asymptomatic phase with physiological or near-physiological motor functions concomitant with a significant reduction in MNs [81,82]. It is worth noticing that the early MNs loss reported in the preclinical model of ALS is relatively stable until the end of the disease, supporting the hypothesis of a therapeutic window to stop disease progression and favour endogenous and/or exogenous compensatory/regenerative mechanisms [82]. We previously reported that reactive gliosis upon selective MNs depletion is characterized by a significant increase of CX43 and intercellular communication [28]. Such a phenomenon activates a positive-loop conditioning spinal ventral horn microenvironment affecting spared MNs compensatory potential via homo-cellular (microglia–microglia or astrocyte–astrocyte) or hetero-cellular (microglia–astrocyte) cross-talk [81]. Microglia during neuroinflammatory and degenerative diseases represents a master regulator of neurons and astrocyte functions via HCs- and GJs-mediated microenvironmental conditioning, releasing excitotoxic stimuli, reactive oxygen species, glutamate, and ATP, thus inducing neuronal distress and cell death [83,84,85,86,87].

In an effort to find potential complementary therapy and pharmacological agents able to revert or to reduce the GJA1/CX43-mediated biological cascade, we analyzed GSPC/GSNC-GJA1 signatures using L1000FWD. This set of evidence aimed at identifying specific FDA approved drugs able to revert the GJA1 transcriptomic profile and to counteract ALS progression. We were able to identify three drugs: amlodipine, sertraline, and prednisolone. To the best of our knowledge, none of these drugs have been tested for their efficacy in ALS models. Amlodipine is a calcium channel blocker and holds antioxidant properties and an ability to enhance the production of nitric oxide [88]. It has been reported that amlodipine mediates a significant reduction in neuronal loss in an experimental model of middle cerebral artery occlusion [89]. The increased circulating levels of transforming growth factor β1 (TGF-β1), found in depressed patients treated with selective reuptake inhibitors, such as sertraline, encouraged the evaluation of sertraline as a potential neuroprotective and anti-inflammatory agent in AD and PD [90,91]. TGF-β1 is an anti-inflammatory cytokine that has been reported to exert protective mechanisms in chronic neuroinflammation and degenerative disease [92]. Finally, the methylated form of prednisolone is often used in relapses of multiple sclerosis and immune system disorders, even if it does not slow the overall progression of the disease [93,94,95,96]. The present study is limited by the lack of an experimental validation and by the potential heterogeneity between datasets. We tried to assess both these limitations contextualizing our finding with previously published experimental evidence and using z-score to reduce variability between samples and between datasets. Such a method is considered a reliable procedure of analysis and can be considered a state-of-the-art method to assess multiple datasets, as demonstrated by previously published literature [97,98,99,100,101,102,103,104,105,106,107]. At the same time, our work provides a valuable overview of the dynamic influence that GJA1/CX43 exerts on MNs depletion that characterizes ALS, highlighting both its diagnostic and therapeutic potential to counteract such a dismal disease.

## 5. Conclusions

The evidence presented in this manuscript shows that substantial differences exist in the cellular brain profile of ALS patients according to the GJA1/CX43 transcripts. However, it should be recalled that this is an exploratory study based on the dataset analysis and more in-depth protein studies are needed to confirm these preliminary results in order to establish the exact role played by the GJA1/CX43 in the spinal cord of patients with ALS. Further studies evaluating the therapeutic potential of drugs able to revert typical GJA1/CX43 signature in ALS patients are needed and may represent a potential therapeutic approach for such a dismal disease.

## Figures and Tables

**Table 1:** Datasets selected.

N°	Dataset	Platform	Org	Samples	Sample Origin	Platform	NDC	ALS	Ref
1	GSE26927	Microarray	Human	19	Spinal cord	GPL6255	9	10	[31]
2	GSE76220	RNA-seq	Human	22	Spinal cord MNs	GPL9115	9	13	[32]

Org: organism; NDC: control not ALS affected; ALS: sporadic amyotrophic lateral sclerosis; MNs: motoneurons.

**Table 2:** Genes correlated to GJA1 in ALS patients.

Samples	Genes	R
Unique Genes significantly positively correlated (GSPC) to GJA1	2542	0.50 < R < 0.91
Unique Genes significantly negatively correlated (GSNC) to GJA1	3110	0.50 < R < 0.96

**Table 3:** Neuro-immune cells signature.

Signatures	N°	Cells and Processes	Source	Unique Genes
Neural cells	1	Astrocyte	GSE67835	177
	2	Microglia	GSE67835	93
	3	Neuron	GSE67835	974
	4	Oligodendrocyte	GSE67835	95
	5	Pericyte immune activated	GSE46236	206
Immune cells	6	M1 macrophages	GSE5099	674
	7	M2 macrophages	GSE5099	132
	8	Natural killer (KN)	GSE22886	114
	9	TH1	GSE22886	191
	10	TH2	GSE22886	85
	11	Endothelial cells	GSE67835	55
	12	CTLs	GSE22886	62
Biological processes	13	Brain microvessels	GSE22886	291
	14	Host virus interaction	KEGG	279
	15	Inflammatory response	KEGG	704
	16	Synaptic transmission genes	GO:0098814	45
	17	Synaptic vesicle cycle	KEGG	78

**Table 4:** Potential anti-ALS drugs identified by the L1000FWD analysis.

N°	Drugs	Score Similarity	Z-Score	Combined Score (CS)	Mechanism of Action (MOA)	Indication(s)
1	Amlodipine	−0.026	1.84	−7.49	Calcium channel blocker	Hypertension
2	Sertraline	−0.021	1.78	−1.83	Selective serotonin reuptake inhibitor	Depression; Obsessive–compulsive disorder; panic disorder; post-traumatic stress disorder
3	Prednisolone	−0.021	1.81	−1.57	Synthetic glucocorticoid with anti-inflammatory and immunomodulatory effect	Adrenergic agent; anti-inflammatory drug; antineoplastic agent; immunosuppressive agent

## Data Availability

The datasets analyzed during the current study are available in the GEODataset repository, Home-GEODataset-NCBI (nih.gov).

## Supplementary Materials

The following supporting information can be downloaded at: https://www.mdpi.com/article/10.3390/biomedicines10092246/s1, Supplementary Table S1.

## Abbreviations

AD	Alzheimer’s disease
ALS	amyotrophic lateral sclerosis
AUC	area under the ROC curve
CNS	central nervous system
CSC	cervical spinal cord
CX	connexin
FDR	false discovery rate
GDA	genomic deconvolution analysis
GEO	gene expression omnibus
GJ	gap junction
GO	gene ontology
GSNC	genes significant negative correlated
GSNC-GJA1	genes significantly negatively correlated to GJA1
GSPC	genes significantly positively correlated
GSPC-GJA1	genes significantly positively correlated to GJA1
HC	hemichannel
LCM	laser capture microdissection
MD	microarray datasets
MeV	MultiExperiment Viewer
MNs	motoneurons
MOA	mode of action
MS	multiple sclerosis
NDC	not demented control subjects–healthy individuals
PD	Parkinson’s disease
SDEG	significantly different expressed genes
